# COVID-19 Outbreak at an Overnight Summer School Retreat ― Wisconsin, July–August 2020

**DOI:** 10.15585/mmwr.mm6943a4

**Published:** 2020-10-30

**Authors:** Ian W. Pray, Suzanne N. Gibbons-Burgener, Avi Z. Rosenberg, Devlin Cole, Shmuel Borenstein, Allen Bateman, Eric Pevzner, Ryan P. Westergaard

**Affiliations:** ^1^Wisconsin Department of Health Services; ^2^Epidemic Intelligence Service, CDC; ^3^Department of Pathology, Johns Hopkins University Baltimore, Maryland; ^4^Department of Medicine, University of Wisconsin School of Medicine and Public Health, Madison, Wisconsin; ^5^Lander College of Arts and Sciences, Tuoro College, New York, New York; ^6^Wisconsin State Laboratory of Hygiene; ^7^CDC COVID-19 Response Team.

During July 2–August 11, 2020, an outbreak of coronavirus disease 2019 (COVID-19) occurred at a boys’ overnight summer school retreat in Wisconsin. The retreat included 152 high school-aged boys, counselors, and staff members from 21 states and territories and two foreign countries. All attendees were required to provide documentation of either a positive serologic test result* within the past 3 months or a negative reverse transcription–polymerase chain reaction (RT-PCR) tests result for SARS-CoV-2 (the virus that causes COVID-19) ≤7 days before travel, to self-quarantine within their households for 7 days before travel, and to wear masks during travel. On July 15, the Wisconsin Department of Health Services (WDHS) began an investigation after being notified that two students at the retreat had received positive SARS-CoV-2 RT-PCR test results. WDHS offered RT-PCR testing to attendees on July 28 and serologic testing on August 5 and 6. Seventy-eight (51%) attendees received positive RT-PCR results (confirmed cases), and 38 (25%) met clinical criteria for COVID-19 without a positive RT-PCR result (probable cases). By the end of the retreat, 118 (78%) persons had received a positive serologic test result. Among 24 attendees with a documented positive serologic test result before the retreat, all received negative RT-PCR results. After RT-PCR testing on July 28, WDHS recommended that remaining susceptible persons (asymptomatic and with negative RT-PCR test results) quarantine from other students and staff members at the retreat. Recommended end dates for isolation or quarantine were based on established guidance (*1**,**2*) and determined in coordination with CDC. All attendees were cleared for interstate and commercial air travel to return home on August 11. This outbreak investigation documented rapid spread of SARS-CoV-2, likely from a single student, among adolescents and young adults in a congregate setting. Mitigation plans that include prearrival quarantine and testing, cohorting, symptom monitoring, early identification and isolation of cases, mask use, enhanced hygiene and disinfection practices, and maximal outdoor programming are necessary to prevent COVID-19 outbreaks in these settings (*3*,*4*).

## Investigation and Findings

Students and staff members (two teachers, one principal, and one emergency medical technician) traveled from 21 states and territories and two foreign countries to attend a faith-based educational retreat for boys in grades 9–11. In an effort to prevent introduction of COVID-19, all attendees were required to provide documentation of either a positive serologic test result within the past 3 months or a negative SARS-CoV-2 RT-PCR result ≤7 days before travel, to self-quarantine within their households for 7 days before travel, and to wear masks during travel. At the retreat, students and counselors were not required to wear masks or social distance, and students mixed freely. Classes were held in outdoor pavilions with approximately 20 students per class seated <6 feet (<2 m) apart at tables. Teachers wore masks during class and were socially distanced from students at all times. The 127 students resided in dormitories (four to six per room) and yurts (eight per room), organized by grade. Beds in dormitory rooms and yurts were tightly spaced with three to four sets of bunks each, shared bathrooms, and shared common areas. Counselors (21; aged 17–24 years) roomed together in dormitories and yurts, and the four staff members resided in four separate housing units.

On July 2, students traveled by air and ground to a regional hub, met with counselors and staff members, and boarded three buses to the retreat (Figure). On July 3, a ninth-grade student (the index patient) who had received a negative RT-PCR result <1 week earlier experienced sore throat, cough, and chills, and received a positive RT-PCR result on July 5. This student later learned that a family member received a positive RT-PCR result approximately 1 week after his departure. At the retreat, he was isolated in a private room, and 11 of his close contacts (including four roommates) were quarantined together in a separate dormitory. The 11 contacts received negative rapid SARS-CoV-2 antigen results and were released from quarantine on July 7, but neither the tests that were conducted nor the results could be verified by public health. During July 4–7, six of 11 close contacts of the index patient and 18 additional students with unknown exposure histories reported new onset of mild symptoms. These students were given masks, but contact tracing was not done and the students were not isolated. On July 13, a second student (one of the 11 initial close contacts of the index patient) received a positive RT-PCR test result at a local clinic. On July 15, WDHS was notified and initiated an outbreak investigation. WDHS instructed retreat organizers in mitigation measures such as symptom monitoring, isolation of symptomatic attendees, and quarantine of contacts, but the capacity for such measures was exceeded by the large volume of symptomatic attendees.

**FIGURE:**
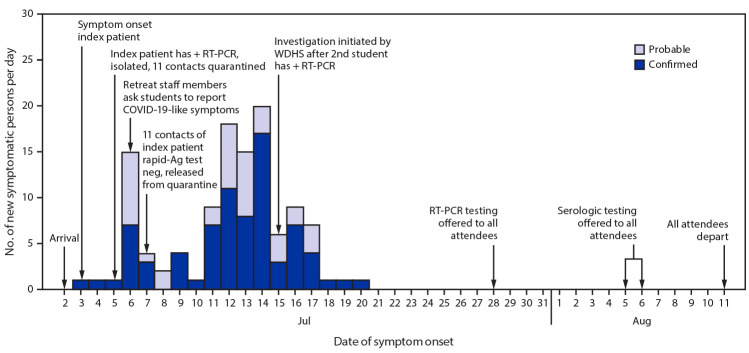
Dates of symptom onset of confirmed (n = 78) and probable (n = 38) COVID-19 cases at an overnight summer school retreat ― Wisconsin, July 2–August 11, 2020 **Abbreviations:** + = positive; Ag = antigen; COVID-19 = coronavirus disease 2019; neg = negative; RT-PCR = reverse transcription–polymerase chain reaction; WDHS = Wisconsin Department of Health Services.

On July 28, WDHS coordinated RT-PCR testing for 148 (97%) of 152 retreat attendees. At the time of specimen collection, no new illnesses had occurred since July 20. During August 5–6, WDHS returned to collect a serum sample for serologic^†^ testing from 148 (97%) attendees; 145 (95%) attendees received both tests. Positive RT-PCR isolates with sufficient cycle threshold values (six of 82; 7%) were analyzed with whole genome sequencing.^§^

A confirmed COVID-19 case was defined as receipt of a positive SARS-CoV-2 RT-PCR test result after July 2 in a retreat attendee. A probable case was an illness meeting clinical criteria for COVID-19 (*5*) with symptom onset during the retreat in an attendee with no prior serologic results who was either not tested by RT-PCR or received a negative RT-PCR result on a sample obtained ≥10 days after symptom onset (to account for attendees who might have cleared the virus by the time of RT-PCR specimen collection). Serologic results were not used for case classification. All analyses were performed using Stata (version 14.2; StataCorp). Fisher’s exact test was used for attack rate comparisons; p-values <0.05 were considered statistically significant. This investigation was reviewed by WDHS for human subjects’ protection and determined to be nonresearch.

Among 152 attendees, 116 (76%) were classified as having confirmed (78; 51%) or probable (38; 25%) COVID-19. Thirty-four (89%) attendees with probable COVID-19 received negative RT-PCR test results on specimens obtained 11–22 days (median = 16 days) after symptom onset. Among the 148 attendees who underwent serologic testing at the end of the retreat (four attendees refused testing), 118 (80%) received positive results. This included 30 (81%) of 37 attendees with probable COVID-19 (one missing), 65 (86%) of 76 with confirmed COVID-19 (two missing), 16 (70%) of 23 attendees with positive serology before the retreat (one missing), and seven (58%) of 12 attendees without a COVID-19 diagnosis or prior serologic result (Table 1). Whole genome sequences for RT-PCR–positive isolates from six attendees differed by 0–1 single nucleotide polymorphisms, suggesting a common source for these six attendees.

**TABLE 1:** Symptoms and serologic test results among persons with confirmed and probable COVID-19 at an overnight summer school retreat ― Wisconsin, July–August 2020

Characteristic	No. (%)		
	All cases	Confirmed*	Probable*
**Total no.**	**116**	**78**	**38**
**Positive serologic result^†^ on Aug 5 or 6, no./total no. (%)^§^**	**95/113 (84)**	**65/76 (86)**	**30/37 (81)**
**Days between symptom onset and serum collection (median, range)**	**23 (16–33)**	**23 (16–33)**	**24 (19–30)**
**Signs/Symptoms**			
None (asymptomatic), no./No. (%)	1/116 (1)	1/78 (1)	NA
Shortness of breath	14 (12)	11 (14)	3 (8)
Cough	85 (73)	53 (68)	32 (84)
Fever	62 (53)	43 (55)	19 (50)
Chills	81 (70)	54 (69)	27 (71)
Sore throat	87 (75)	57 (73)	30 (79)
Fatigue	92 (79)	60 (77)	32 (84)
Myalgia	54 (47)	31 (40)	23 (61)
Loss of taste or smell	55 (47)	40 (51)	15 (39)
Diarrhea	32 (28)	19 (24)	13 (34)
Nausea or vomiting	39 (34)	25 (32)	14 (37)
Headache	96 (83)	64 (82)	32 (84)
Congestion or runny nose	86 (74)	58 (74)	28 (74)

**Abbreviations:** COVID-19 = coronavirus disease 2019; NA = not applicable; RT-PCR = reverse transcription–polymerase chain reaction.

* Confirmed: positive RT-PCR test results; probable: met clinical criteria only.

^†^ Abbott Architect SARS-CoV-2 Immunoglobulin G chemiluminescent microparticle assay.

^§^ Four attendees refused testing. An additional 16 of 23 attendees with prior positive serologic results and seven of 12 without COVID-19 diagnoses or prior positive serologic results received positive serologic test results.

At least one confirmed case occurred in every dormitory room and yurt (Supplementary Figure, https://stacks.cdc.gov/view/cdc/95625). Attack rates did not differ significantly among counselors and students, dormitories and yurts, or grade levels (Table 2). All four staff members received negative RT-PCR test results; one staff member (an emergency medical technician) was classified as having a probable case. To comply with the retreat’s attendance requirements, 24 (16%) attendees provided documentation of a positive serology results before the retreat. All 24 received negative RT-PCR results. Six (25%) experienced mild symptoms at the retreat but were not classified as having confirmed or probable COVID-19. Excluding the 24 attendees with previous positive serologic results, the COVID-19 attack rate on the remaining susceptible population was 91% (116 of 128). One (1.2%) of 78 persons with a positive SARS-CoV-2 RT-PCR test result was asymptomatic. All illnesses were mild to moderate, and no hospitalizations or deaths occurred. 

**TABLE 2:** Characteristics of persons with confirmed or probable COVID-19 among students, counselors, and staff members at an overnight summer school retreat (N = 152), by case classification status ― Wisconsin, July–August 2020

Characteristic	No./total no. (%)		
	All cases	Confirmed*	Probable*
**Role (age range, yrs) [no. of persons]**			
**Students, grade**			
9 (14–15) [42]	34/42 (81)	23/40 (58)^†^	11/42 (26)
10 (15–16) [45]	32/45 (71)	24/43 (56)^†^	8/45 (18)
11 (16–17) [40]	34/40 (85)	17/40 (43)	17/40 (43)
All students [127]	100/127 (79)	64/123 (52)^†^	36/127 (28)
**Counselors (17–24) [21]**	15/21 (71)	14/21 (67)	1/21 (5)
**Staff members (21–45) [4]**	1/4 (25)	0/4 (0)	1/4 (25)
**Total [152]**	**116/152 (76)**	**78/148 (53)**	**38/152 (25)**
**Room type**			
Dormitory [94]	71/94 (76)	53/90 (59)	20/94 (21)
Yurt [54]	44/54 (82)	25/54 (46)	19/54 (35)
Staff member housing [4]	1/4 (25)	0/4 (0)	1/4 (25)
**Previous serologic results (IgG)**			
Positive [24]	0/24 (0)	0/24 (0)	0/24 (0)
None documented [128]	116/128 (91)	78/124 (63)^†^	38/124 (31)

**Abbreviations:** COVID-19 = coronavirus disease 2019; IgG = immunoglobulin G; RT-PCR = reverse-transcription–polymerase chain reaction.

* Confirmed: positive RT-PCR test results; probable: met clinical criteria only.

^†^ Four students did not have RT-PCR test results (two were not present during specimen collection and for two others, laboratory errors occurred).

## Public Health Response

When WDHS initiated the investigation on July 15, retreat staff members reported that the majority of students had recovered from mild illnesses. After RT-PCR testing on July 28, WDHS recommended that remaining 36 susceptible persons (24%; asymptomatic and with negative RT-PCR test results) quarantine from other students and staff members at the retreat. Recommended end dates for isolation or quarantine were based on established guidance (*1**,**2*) and determined in coordination with CDC’s Division of Global Migration and Quarantine. Outdoor coursework and recreational programming were able to continue for the duration of the retreat, and all attendees were cleared for interstate and commercial air travel to return home on August 11.

## Discussion

Extensive and rapid transmission of SARS-CoV-2 occurred at an overnight retreat where adolescents and young adults aged 14–24 years had prolonged contact and shared sleeping quarters. A single student, who received a negative SARS-CoV-2 RT-PCR test result <1 week before the retreat and experienced symptoms 1 day after arriving, was the likely source of introduction, resulting in infection of 76% of attendees. Similar rapid spread has been described among younger children in overnight camps (*6*,*7*) and adults in congregate settings (*8*,*9*).

Nonpharmaceutical interventions have been effective in preventing SARS-CoV-2 transmission at overnight camps (*3*). Effective measures include prearrival quarantine and testing, cohorting, symptom monitoring, physical distancing, mask use, enhanced hygiene measures, enhanced cleaning and disinfection, outdoor activities and programming, and early identification of infections and isolation. At this retreat, organizers required documentation of a negative prearrival RT-PCR result, 7-day prearrival quarantine, and outdoor programming, but did not implement other recommended nonpharmaceutical interventions. The capacity of retreat organizers to contain transmission through isolation and quarantine early in the outbreak was exceeded given the large number of persons exposed and experiencing symptoms. A robust COVID-19 mitigation plan that included a full 14-day prearrival quarantine might have prevented introduction of SARS-CoV-2 in this setting. As well, cohorting of attendees for 14 days after arrival might have permitted early containment of the outbreak. Finally, earlier engagement with public health authorities to discuss recommended mitigation strategies (*4*) might also have aided prevention and control efforts.

An important feature of this outbreak was that 24 attendees had documented evidence of antibodies to SARS-CoV-2 before arrival. None of these persons received a positive SARS-CoV-2 RT-PCR test result at the retreat. Evidence to date is insufficient to determine whether the presence of detectable antibodies indicates protective immunity^¶^ or how long such immunity might persist. The absence of RT-PCR–confirmed infections among persons with previous positive serology results suggests that some protective effect was present, given the high attack rate observed at the retreat.

The proportion of SARS-CoV-2 infections that were asymptomatic (1%) in this population was low, compared with those described in other published reports (*10*). Retreat staff members kept detailed symptom logs for students, which likely facilitated improved ascertainment of mild or delayed COVID-19 symptoms, compared with other settings and might explain the low rate of asymptomatic infection observed. In addition, some mild symptoms experienced by attendees possibly were not related to infection (e.g., allergies or travel fatigue) or were caused by another viral illnesses, which would have led to overestimation of the number of probable cases.

The findings in this report are subject to at least four limitations. First, RT-PCR testing was conducted after the outbreak (no new illnesses in the 8 days before testing), likely leading to underestimation of the number of confirmed cases. Second, baseline serology results were not available for all retreat attendees. Some positive results in follow-up serologic testing might have been caused by past undocumented infections rather than SARS-CoV-2 infection at the retreat. Third, dates of prior illnesses among attendees with previous positive serologic results were not known, and the duration of possible acquired immunity against SARS-CoV-2 infection could not be assessed. Fourth, the definition for probable COVID-19 used in this investigation was adapted from the Council of State and Territorial Epidemiologists interim COVID-19 case definition (*5*) to account for the delay in RT-PCR testing and availability of prior serologic results for some attendees; results may not be comparable with other outbreak investigations.

SARS-CoV-2 can spread rapidly among adolescents and young adults in a congregate setting with inadequate COVID-19 mitigation measures. These findings provide preliminary evidence that detectable antibodies might provide protection against new SARS-CoV-2 infections for an unknown duration. A robust COVID-19 mitigation plan developed in collaboration with public health authorities is important for preventing and containing similar outbreaks at overnight camps and residential schools. Avoidance of travel for attendees who were in isolation or quarantine likely prevented transmission to communities and family members during this outbreak and could be considered in COVID-19 mitigation plans for other congregate settings. To prevent introduction of COVID-19, mitigation plans should also include prearrival quarantine, prearrival and postarrival testing and symptom screening, the ability to isolate and quarantine, cohorting, physical distancing, mask use, enhanced hygiene and disinfection, and maximal outdoor programming (*3*).

**Summary**

**What is already known about this topic?**

SARS-CoV-2 can spread rapidly in congregate settings such as overnight camps.

**What is added by this report?**

During July 2–August 11, 2020, a COVID-19 outbreak at an overnight high-school retreat likely began with a single student who had received a negative SARS-CoV-2 molecular test result <1 week before the retreat and led to 116 (76%) diagnosed COVID-19 cases among attendees.

**What are the implications for public health practice?**

A multicomponent COVID-19 mitigation plan including prearrival quarantine and testing, cohorting, symptom monitoring, early identification and isolation of cases, mask use, and enhanced hygiene and disinfection practices is critical for reducing the risk for SARS-CoV-2 transmission in congregate settings such as residential schools and overnight camps.

## Footnotes

* Documentation provided by retreat attendees from a laboratory of any SARS-CoV-2 serologic test done within 3 months before the retreat.

† Abbott Architect SARS-CoV-2 Immunoglobulin G chemiluminescent microparticle assay (Abbott Laboratories). https://www.corelaboratory.abbott/us/en/offerings/segments/infectious-disease/sars-cov-2.

§ Whole genome sequencing conducted by Wisconsin State Laboratory of Hygiene on positive RT-PCR specimens with cycle threshold values less than 30. Samples were extracted on a KingFisher Flex with MagMAX reagents, library preparation was performed following the ARTIC protocol, and sequencing was performed on an Oxford Nanopore Technologies MinION.

¶ https://www.cdc.gov/coronavirus/2019-ncov/lab/resources/antibody-tests-guidelines.html.
